# Increased ETV4 expression correlates with estrogen-enhanced proliferation and invasiveness of cholangiocarcinoma cells

**DOI:** 10.1186/s12935-018-0525-z

**Published:** 2018-02-20

**Authors:** Ekapot Singsuksawat, Chanitra Thuwajit, Komgrid Charngkaew, Peti Thuwajit

**Affiliations:** 1 Graduate Program in Immunology, Faculty of Medicine Siriraj Hospital, Mahidol University, 2 Wang Lang Road, Bangkok Noi, Bangkok, 10700 Thailand; 2 Department of Immunology, Faculty of Medicine Siriraj Hospital, Mahidol University, 2 Wang Lang Road, Bangkok Noi, Bangkok, 10700 Thailand; 3 Department of Pathology, Faculty of Medicine Siriraj Hospital, Mahidol University, 2 Wang Lang Road, Bangkok Noi, Bangkok, 10700 Thailand; 4 NANOTEC-Mahidol University Center of Excellence in Nanotechnology for Cancer Diagnosis and Treatment, Faculty of Medicine Siriraj Hospital, Mahidol University, 2 Wang Lang Road, Bangkok Noi, Bangkok, 10700 Thailand

**Keywords:** Cholangiocarcinoma, ETV4, Estrogen, Tamoxifen, Mouse xenografted model

## Abstract

**Background:**

Cholangiocarcinoma (CCA) is one of the worst prognosis cancer. The survival time of CCA patients is related to serum estrogen levels and estrogen has been found to enhance the proliferation and invasiveness of CCA cells in vitro. This has led to the suggestion that estrogen may play an important role in the progression of CCA. This study tests the relevance of the previous in vitro findings in vivo using a mouse xenograft model of CCA, and investigates possible signaling mechanisms involved.

**Methods:**

KKU-213 and KKU-139 CCA cell lines were used in the experiments, xenografted to nude mice and treated with a potent estrogenic agent, 17β-estradiol (E2), and/or with tamoxifen (TAM), an estrogen antagonist.

**Results:**

The results demonstrated that E2 could accelerate growth of the xenograft-tumor and the effect was inhibited by TAM. PCR array screening of E2 responsive genes suggested ETV4 as a promising candidate intracellular mediator. ETV4-knockdown CCA cells were generated and these showed a diminished responsiveness to E2 in both cell and spheroid proliferation assays, and in invasion tests. These results point to ETV4 as a possible mediator of E2-activated CCA progression and as a potential target of TAM-mediated inhibition.

**Conclusions:**

Finally, TAM may be suggested as an adjunctive treatment of CCA to improve the conventional cytotoxic method with more patient toleration.

**Electronic supplementary material:**

The online version of this article (10.1186/s12935-018-0525-z) contains supplementary material, which is available to authorized users.

## Background

Cholangiocarcinoma (CCA) is a malignant tumor arising from the epithelial lining of the biliary tract, excluding the Ampulla of Vater and the gall bladder [1]. CCA can be divided into intrahepatic CCA (ICC) and extrahepatic CCA (ECC) according to the position of tumor [1]. The cystic duct further serves as a dividing line between perihilar and distal subtypes of ECC. ICC, perihilar ECC and distal ECC all have different etiologies, epidemiology and clinical management [2, 3].

Although the global incidence of CCA is low, accounting for less than 1% of all cancers, (with ECC more common than ICC) [1], it is a common cancer in Thailand, where the majority of CCA cases present as ICC. The latest official report from the Thailand National Cancer Institute, (2010–2012), has shown that the three Thai provinces with the highest incidence of confirmed diagnoses of CCA, Ubon Ratchathani, Khon Kaen and Udon Thani provinces, had average annual age-standardized rates per 100,000 individuals (male/female) of 28/12.7, 15/5.2 and 10/5.1 respectively [4]. In contrast, European crude incidence rates per 100,000 people, of both sexes, were 0.97 for ICC and 1.44 for ECC [1].

Cholangiocarcinoma is a devastating and challenging cancer that results in both difficult diagnosis and bad prognosis [5]. The majority of CCA patients usually remain asymptomatic until the metastatic stage, resulting in a low survival time: patients may die within a year of diagnosis [5–7]. Consequently, chemotherapy and/or radiotherapy usually produce responses that are too poor to warrant their application [6]. The only choice of therapy is surgical resection or liver transplantation, but the outcomes are still poor [7]. The development of alternative management strategies that lead to better outcomes is urgently required and would benefit from a better understanding of CCA tumorigenesis.

Biliary obstruction is commonly found in CCA, and experiments have suggested that this can cause higher estrogen levels through reduced estrogen turnover rate as a consequence of reduced steroid hormone converting enzyme levels [8]. Thus, biliary obstruction may underlie the excessively high levels of estrogen accumulating in CCA patients that have been associated with significantly poorer survival [9, 10]. 17β-estradiol (E2) is the most potent form of natural human estrogens. It primarily functions to regulate the development and physiology of the female reproductive system but it is important in an additional range of physiological processes. It has been shown to be involved in the carcinogenesis of, not only breast [11], endometrial [12] and ovarian [13] cancers, but also non-gynecological cancers, including osteosarcoma [14], prostate cancer [15] and thyroid carcinoma [16]. E2 is the one mediator that is known to stimulate cholangiocyte proliferation and has been considered to be involved in the development and progression of pathologies of the biliary system [17]. In addition, many studies have shown that E2 also stimulates the secretion of certain mediator proteins which have been found to correlate with the progression of CCA, such as insulin-like growth factor 1 [18], interleukin-6 [19], vascular endothelial growth factor [20], and trefoil factor family 1 [9]. Estrogen receptors (ERs) are found in normal biliary epithelial cells, and their expression is increased in CCA cells [9, 17]. Moreover, exogenous estrogens have been considered a risk factor in the carcinogenesis of biliary tree cancer [21]. Thus E2 is a multifunctional hormone, making the identification of intermediate intracellular molecules involved in estrogen associated tumorigenesis challenging. However, understanding this process may lead to new targets for controlling the progression of CCA.

E26 transformation-specific (ETS) variant 4 (ETV4) is a member of the polyomavirus enhancer activator 3 (PEA3) subfamily of ETS transcription factors that play important roles in both normal physiology and in pathological mechanisms [22]. This includes development of malignancies—tumor cell progression, transformation, invasion and metastasis—by activating or repressing the transcription activity of downstream cancer-related target genes [22–24]. ETV4 abnormalities may present either as overexpression of ETV4 or gene fusions. ETV4 overexpression has been implicated in the progression of many types of cancer including esophageal [24], prostate [25] and breast cancer [26]. In addition, genomic and transcriptomic ETV4 gene fusions have been demonstrated in Ewing sarcoma [27] and prostate cancer [28] and suggested to have role in carcinogenesis of both cancer. Analysis of the ETV4 promoter structure and its activity revealed that there are many putative binding sites for a number of transcription factors including PEA3, estrogen-binding subunit and estrogen response elements (EREs), which are activated by ETV4 itself and ERs. This data suggests that ETV4 may have a key role in estrogen-stimulated signaling [29].

In this study, an animal model was used to explore the effect of E2 in CCA progression. E2-induced tumorigenic properties were assayed in CCA cell lines, focusing on the expression of ETV4 as a tentative E2-regulated gene. Finally, ETV4 knockdowns were used to inhibit E2-driven progression of CCA. This study provides a better understanding of the role of E2 in the progression of CCA, highlighting the involvement of specific E2-induced genes. It indicates ETV4 as a possible molecular target which may, in future, prove useful as an additional prognostic marker or in the therapy of CCA patients.

## Methods

### Cell lines and mice

Two different CCA cell lines, including KKU-213 (derived from mixed-differentiated adenocarcinoma tissue of an ICC male patient) [9, 30, 31] and KKU-139 (derived from an adenosquamous cell carcinoma tissue of an ICC male patient) [32] were established and kindly donated by Professor Dr. Banchob Sripa, Department of Pathology, Faculty of Medicine, Khon Kaen University. These cell lines were cultured at 37 °C in a humidified 5% CO_2_ incubator in complete medium: Ham’s F12 nutrient mixture (Invitrogen, Carlsbad, CA, USA) supplemented with 10% fetal bovine serum (FBS; Invitrogen).

Male athymic BALB/c nude mice, aged 3–8 weeks old, were supplied by the National Laboratory Animal Center, Mahidol University. The mice were maintained in sterile bedding and housed in controlled temperature, humidity, and light/dark cycle (12:12 h) according to the Animal Care and Use Protocol approved by Siriraj Animal Care and Use Committee (SI-ACUP 011/2554). Sterile tablet food and water were provided ad libitum.

### Cell lines characterization for E2 and ERs

Both cell lines were measured the base line E2 production. Briefly, 2 × 10^5^ of CCA cells were cultured in 6-well plates using 3 ml of phenol red-free DMEM-F12 (Invitrogen) with 10% charcoal-stripped FBS (Invitrogen) for 24 h. Then condition medium was collected and centrifuged for removing of cell content. E2 level was measured by automated machine following routine service standard protocol of Department of Clinical Pathology, Siriraj hospital.

Estrogen receptor (ER) α and β genes expression of both cell line were measured using RT-PCR. For reverse transcription, total RNA was extracted using the PerfectPure^®^ RNA Cultured Cell Kit (5 PRIME, Gaithersburg, MD, USA), according to the company’s protocol. The total extracted RNA was converted to cDNA by MMLV reverse transcriptase using the SuperScript™ III First-Strand Synthesis System (Invitrogen) according to the company’s protocol. The semi-quantitative expression levels of candidate genes were determined using the LightCycler^®^ 480 system (Roche Applied Science). The relative expression of both genes in CCA cells compared to those in MCF-7 and MDA-MB-231 breast cancer cells was calculated using the company’s 2^−ΔΔCp^ equation relative to the internal control (36B4 ribosomal protein mRNA expression). Primer sequences of both ERs and internal control are shown in Additional file 1: Table S1.

### Estrogen and tamoxifen effects on in vitro CCA cell proliferation and invasion assay

Briefly, to reduce potential estrogenic effects of phenol red on the CCA cell lines, the complete culture medium was changed to phenol red-free DMEM-F12 with 2% charcoal-stripped FBS 1 day before treatment. Twenty-four hours later, cells were treated in with 1 nM E2 (water soluble cyclodextrin-encapsulated 17β-estradiol; Sigma-Aldrich, St. Louis, MO, USA) and/or 10 µM 4-hydroxytamoxifen (TAM) (Sigma-Aldrich) diluted in 2% charcoal-stripped FBS supplemented, phenol red free DMEM-F12 medium.

For proliferation assays, 1000 CCA cells were cultured in 96-well plates and treated with E2 and/or TAM as described. CellTiter 96^®^ AQueous One Solution Cell Proliferation Assay^®^ (Promega, Madison, WI, USA) was used for cell counting according to the company’s protocol. For each treatment condition, cell numbers were counted and medium was changed on days 2, 4 and 6 post-treatment.

Prior to in vitro invasion assays, 24-well BD Biocoat Matrigel™ invasion chambers (Becton–Dickinson, Franklin Lakes, NJ, USA) were prepared following the company’s protocol. After 24 h culture in phenol red free medium, cells were treated with E2 and/or TAM, as described, for the ensuing 24 h. Thereafter, cells were detached using trypsin, and 100,000 CCA cells were placed in the upper chamber of each well. Cells were then further treated with E2 and/or TAM for another 6 h, before the Matrigel and cells on the top side of the upper chamber were removed. The invading cells that attached to the underside of the upper chamber were fixed with 40% (v/v) methanol for 30 min, then stained with haematoxylin. The total numbers of invading cells per field were counted using an inverted microscope, and calculated as fold invasion in comparison with untreated control wells.

### Spheroid proliferation assay

Proliferation of tumor cells was also determined in three dimensional (3D) model using spheroid proliferation assay. Into each well of a pre-cooled 96-well ultra-low attachment plate (Corning, Amsterdam, NL), 2 × 10^3^ KKU-213 or KKU-139 CCA cells were seeded in 200 µl of medium. Cell suspensions were supplemented with 2.5% cold Matrigel™ (Becton–Dickinson) and centrifuged at 1000 rpm, 4 °C, for 3–5 min to facilitate cell–matrix interaction. Cultures were then incubated at 37 °C for 96 h to allow spheroid initiation. The end of this initial period was labelled Day 0. From that time onwards, treatment with E2 and/or TAM began and media were changed every 2 days. Image capture and measurement of mean spheroid diameter was performed at day 4, 7, 10, 13 and 15. Mean diameter was converted to radius (r), and then to volume of tumor spheroid using the formula 4/3πr^3^.

### Polymerase chain reaction (PCR) array

RT^2^ Profiler™ PCR Array Human Tumor Metastasis (Cat. No. PAHS-028A) (QIAGEN, Valencia, CA, USA) was performed as per the company’s protocol for the purpose of screening for estrogen-regulated, metastasis-related, target molecules. Briefly, following the reverse transcription (RT) step, total RNA from either E2-treated, or untreated control, KKU-213 CCA cells was extracted then converted to first strand cDNA using RT^2^ First Strand Kit (Qiagen). Next, the cDNA template was mixed with RT^2^ qPCR Master Mixes (Qiagen) and the mixture was aliquoted into each well of the plate containing pre-dispensed gene-specific primer sets. After that, real-time PCR was performed using the LightCycler^®^ 480 system (Roche Applied Science, Mannheim, Germany), the crossing point (Cp) was recorded and the relative expression of each gene was determined by 2^−ΔΔCp^ equation as specified in the manufacturer’s instructions.

### Expression level of targeted molecules in CCA cells by RT-real time PCR

Target genes were chosen from the PCR array results. The relative expression of selected genes from samples of each in vitro or in vivo treatment group was measured by RT-PCR. The relative expression of target genes in CCA cells treated with E2 and TAM compared to those in untreated control cells was calculated using the company’s 2^−ΔΔCp^ equation relative to the internal control (36B4). Primer sequences of target genes are shown in Table 1.

**Table 1:** Primer sequences for gene expression analysis

Gene	Accession no.		Primer sequence (5′ → 3′)	Size (bp)
*36B4*	NM_001002.3	F R	CTTCCCACTTGCTGAAAAG CCAAATCCCATATCCTCGT	168
*ETV4*	NM_001986.2	F R	GTCACTTCCAGGAGACGTGG ATAGGCACTGGAGTAAAGGCAC	218

### Green fluorescence protein (GFP) labeling

LeGO-G2 vector, which encodes green fluorescent protein (GFP), was kindly provided by Dr. Bunpote Siridechadilok, National Center for Genetic Engineering and Biotechnology (BIOTEC), National Science and Technology Development Agency, Ministry of Science and Technology, Thailand. Detailed information on vector sequences and production procedures is provided on the LeGO vector website (http://www.lentigo-vectors.de). In brief, and in accordance with the standard protocol, CCA cells were seeded at 2 × 10^5^ cells per well in 6-well plates in complete medium. For transduction, polybrene was added to a final concentration of 8 µg/ml in supernatant containing vector and thoroughly mixed. Cells were then incubated for 24–48 h and then the media were replaced with Opti-MEM^®^ (Invitrogen). GFP protein expression in cells was examined under a fluorescence microscope. The successfully transfected cells were further maintained in their regular medium.

### Tumor injection and treatment in mice

Mice were injected subcutaneously in the right frank with 4 × 10^6^ cells of GFP-transfected CCA cell lines and received their respective treatments with their food every day for 2 weeks: the control group was fed the regular diet with no treatment; the E2-treated group received tablet food supplemented with E2 (2.7 mg/kg/day) [33]; the TAM-treated group received tablet food supplemented with Tam (40 mg/kg/day) [34], and the E2 + TAM treated group received tablet food supplemented with E2 and TAM (at the same dosage as the E2 and Tam treated groups). Rate of food intake by each mouse was monitored. After 2 weeks, all mice were sacrificed by cardiac puncture to collect blood serum for measuring E2 levels, and tumor weights were measured. Tumor masses and metastasis were observed using in vivo imaging (Carestream In Vivo Imaging System FX Pro^®^; Bruker, Billerica, MA, USA). Tumor nodules were separately collected in RNAlater^®^ RNA stabilization reagent (QIAGEN) for performing RNA extraction, and in 10% formaldehyde for paraffin-embedded tissue block preparations for immunohistochemistry.

### Immunohistochemistry

Both CCA cell lines were prepared for immunohistochemistry by seeding 10^5^ cells per well in 6-well plates and treated with E2 or TAM as described. After treatment, cells were collected and centrifuged to form a pellet which was then prepared as a paraffin-embedded cell block. Both cell blocks and tissue blocks were sliced and stained using the following immunoperoxidase method. The immunodetection was performed using monoclonal anti-ETV4 1:500 (ab70425, Abcam, Canbridge, MA, USA) as the primary antibody, incubated for 1 h at room temperature. Mouse Envision + SystemTM horseradish peroxidase (HRP)-labeled polymer (Dako, Agilent Technologies, Santa Clara, CA, USA) was used for 30 min at room temperature as the secondary antibody. After that, 3,3′-diaminobenzidine (DAB) was used for color development. A slide scanner (Aperio Scanscope^®^, Leica Microsystems, Wetzlar, Germany) was used to generate image files. The level of expression of ETV4 in cell blocks was derived from the percentage of positive cells with stained nuclei using the default setting of the Aperio Imagescope Viewer Software^®^ (version 12.03.002 with Nuclear_v9 algorithm, Leica Microsystems). The percentage of positive nuclei staining in each treatment group was compared to the control.

### Short hairpin RNA (shRNA) plasmid transfection

The SureSilencing shRNA plasmid, GFP^®^ (QIAGEN) system was chosen for knocking down genes of interest. Target shRNA plasmids and negative control shRNA vectors were transfected into CCA cells using Lipofectamine 3000^®^ (Invitrogen) in accordance with the manufacturer’s instructions. The mixture was added to the cells, incubated for 24 h before the transfected cells were visualized by fluorescence inverted microscope. To evaluate the effectiveness of the knockdown procedure, RNA was harvested 48–96 h post-transfection and tested using RT-real time PCR. Knockdown cells were further checked for their tumorigenic functions in response to E2.

### Statistical analysis

All quantified data was presented as average ± standard deviation (SD) and representative of at least two independent experiments. Statistics on the effect of estrogen on CCA progression in both in vitro and in vivo experiments were carried out using either Student’s t test or Mann–Whitney Rank Sum test to compare treatments. A *P* value of less than 0.05 was considered statistically significant.

## Results

### E2 production and ERs expression in CCA cell lines

After cultures for 24 h in 3 ml of media, 2 × 10^5^ cells of KKU-213 CCA cell lines could produce E2 to reach 0.033 and 0.018 nM for KKU-139. ERs expressions of both CCA cell lines were measured by RT-real time PCR and compared with MCF-7 breast cancer cells, a well-known ERα positive cell, and MDA-MB-231, a triple negative breast cancer cell line. The results were shown in Additional file 1: Figure S1.

### In vitro effect of estrogen on tumorigenic properties of CCA cell lines

**Fig. 1:**
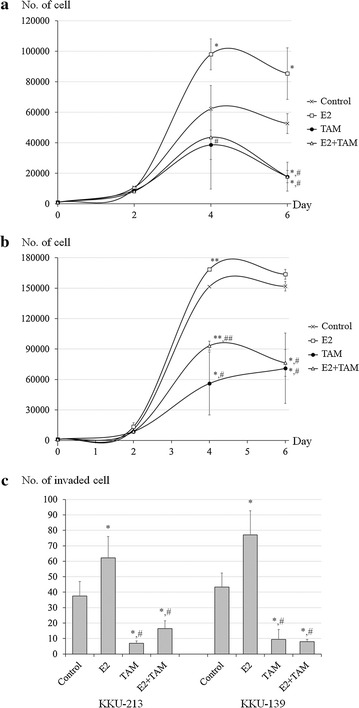
*In vitro* effect of estrogen on tumorigenic properties of CCA cell lines. Cell proliferation of **a** KKU-213 and **b** KKU139 in response to E2 and/or TAM treatment in vitro. Cell numbers were measured on days 2, 4 and 6 and were calculated using an MTS standard for each cell line. Arrows indicate the variables compared for statistical significance. **c** In vitro invasion assay of KKU-213 and KKU-139 CCA cells stimulated by E2 and/or TAM. Experiments were performed as triplicated experiments. Symbol * and ** determined statistically significant difference compared to untreated control group with *P* < 0.05 and < 0.001, respectively. Symbol # and ## determined statistically significant difference compared to E2 treated group with *P* < 0.05 and < 0.001, respectively

The effect of estrogen on the proliferation and invasiveness of CCA cells was studied using the KKU-213 and KKU-139 CCA cell lines. Cell proliferation in response to E2 (1 nM) and/or TAM (10 µM) was measured in a 3-(4,5-dimethylthiazol-2-yl)-5-(3-carboxymethoxyphenyl)-2-(4-sulfophenyl)-2H-tetrazolium (MTS)-based assay. E2 significantly stimulated cell proliferation in both cell lines, an effect which was inhibited by TAM (Fig. 1a, b). E2 also significantly enhanced the invasiveness of both CCA cell lines, (approximately 1.7 times for KKU-213 and 1.8 times for KKU-139), and this E2-stimulated increase was also inhibited by TAM (Fig. 1c).

**Fig. 2:**
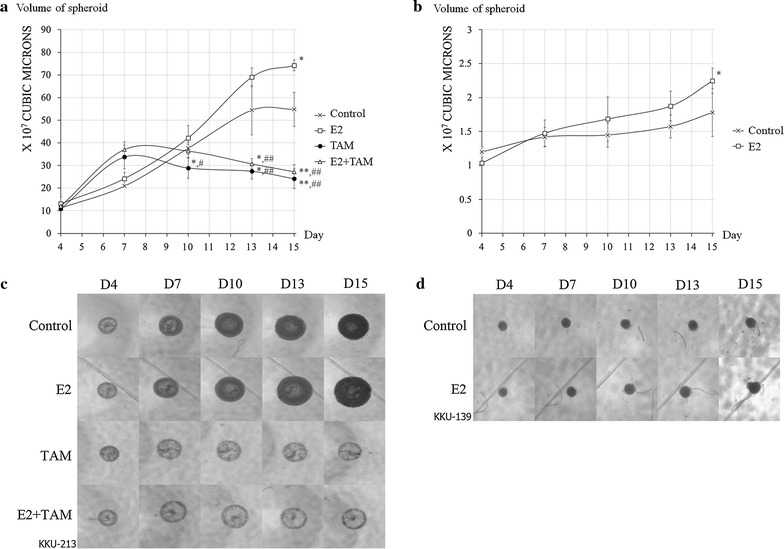
Estrogen stimulated proliferation of CCA cell lines in a 3D system. **a** Diagrammatic growth curve of KKU-213. **b** Diagrammatic growth curve of KKU-139. **c** Representative picture of KKU-213 spheroids. **d** Representative picture of KKU-139 spheroids. Experiments were performed as quadruplicated experiments. Symbol * and ** determined statistically significant difference compared to untreated control group with *P* < 0.05 and < 0.001, respectively. Symbol # and ## determined statistically significant difference compared to E2 treated group with *P* < 0.05 and < 0.001, respectively

Proliferation assays in a 3D system showed statistically significant results similar to those obtained in the cell proliferation assay for both cell lines (Fig. 2a, b). However, there are no results for KKU-139 receiving either TAM or E2 + TAM as this cell line did not form spheroids under these treatment conditions. Moreover, the spheroid size of KKU-139 receiving other treatments was markedly smaller than that of KKU-213. Representative spheroids for all conditions are shown in Fig. 2c, d.

### Effect of estrogen on CCA progression in nude mouse

**Fig. 3:**
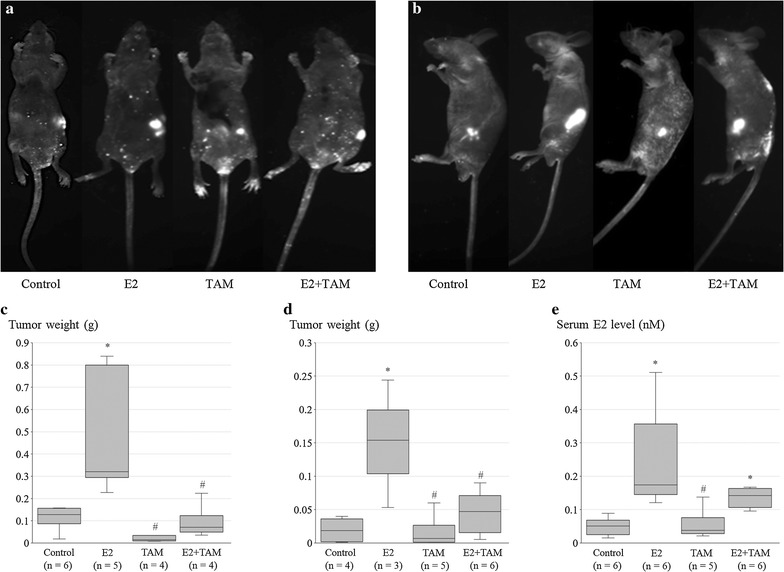
Estrogen-stimulated CCA cell line proliferation in vivo. GFP-tagged primary tumor masses are visualized using excitation/emission wave length as 488/510 nm. Tumors presented as the brightest spot in the right flank of mice xenografted with **a** KKU-213 or **b** KKU-139 and treated with E2 and/or TAM as indicated. Weight of primary tumors extracted from mice xenografted with **c** KKU-213 or **d** KKU-139 and treated as indicated. **e** Serum E2 concentration in each treatment group. Number of mice (n) in each group was determined in diagrams. Symbol * determined statistically significant difference compared to untreated control group with *P* < 0.05. Symbol # determined statistically significant difference compared to E2 treated group with *P* < 0.05

Male athymic BALB/c nude mice were subcutaneously xenografted with GFP-transfected KKU-213 or KKU-139 CCA cells and then E2, Tam, or both were administered orally for 2 weeks. Tablet food intake was monitored during this period and was not significantly different between the treatment groups (data not shown). During the experiment, the fluorescence of tumor masses (Fig. 3a, b) was observed using in vivo imaging. However, metastasis was not detected by this system, and the few mice that displayed metastasis after sacrifice had a very small primary mass and had been lethargic prior to sacrifice (data not shown); therefore, these animals with metastasis were excluded from comparisons of the primary mass. There were 3–6 mice per group. After sacrifice, the tumor mass was extracted and weighed (Fig. 3c, d). The results from mice xenografted with either CCA cell line showed a significant increase in growth of the primary tumor nodule in mice treated with E2 compared to control groups. TAM significantly inhibited this phenomenon, although when administered alone it produced no significant effect in comparison to control groups. Serum E2 concentrations were also confirmed to be higher in the E2-treated groups (E2 and E2 + Tam groups) (Fig. 3e).

### Screening and validating target estrogen-regulated gene in CCA

**Fig. 4:**
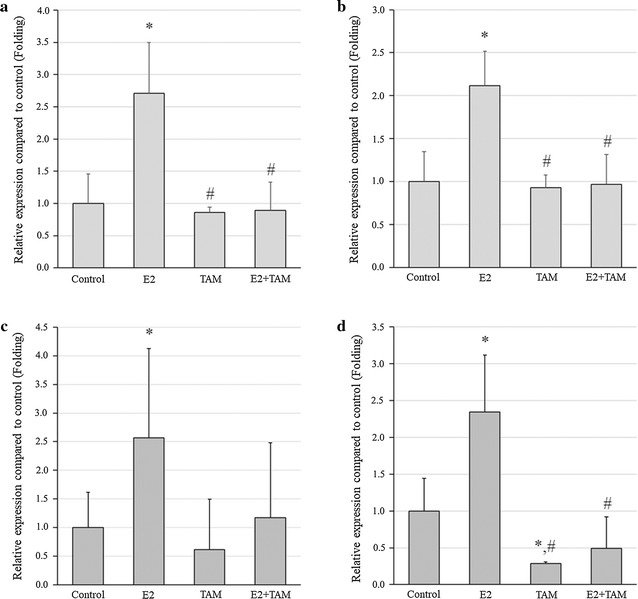
Relative expression of ETV4 mRNA in CCA cell lines treated with E2 and/or TAM compared to untreated control: **a** KKU-213 treated in vitro; **b** KKU-139 treated in vitro; **c** KKU-213 xenografted tumor masses; **d** KKU-139 xenografted tumor masses. Experiments were performed as triplicated experiments. Symbol * determined statistically significant difference compared to untreated control group with *P* < 0.05. Symbol # determined statistically significant difference compared to E2 treated group with *P* < 0.05

Control and E2-treated KKU-M213 CCA cells were assayed using RT^2^ Profiler PCR Arrays in order to identify E2-regulated genes. Among a total of 84 genes analyzed, the relative expression of 14 genes was up-regulated (by more than twofold) in response to estrogen, while 9 genes were downregulated (by less than 0.5-fold) (Table 2). Of these, ETV4 was of particular interest due to its known function, so the response of ETV4 to estrogen treatment was further examined in both CCA cell lines, in both in vitro and in vivo systems, using real time PCR. In both CCA cell lines in vitro, ETV4 gene expression was significantly enhanced by E2 in comparison to the untreated control, while TAM treatment reduced it (Fig. 4a, b). Levels of ETV4 expression in the xenografted tumor masses of mice bearing KKU-213 and KKU-139 were also significantly increased in the E2 group and diminished in cells from mice treated with TAM (Fig. 4c, d).

**Table 2:** E2 targeted genes from RT^2^ Profiler™ PCR array human tumor metastasis compared between E2-treated KKU-213 and untreated control

Genes	Full name	Fold change
Up-regulated gene		
ITG B3	Integrin, beta 3 (platelet glycoprotein IIIa, antigen CD61)	15.81
MET	Met proto-oncogene (hepatocyte growth factor receptor)	7.38
MMP11	Matrix metallopeptidase 11 (stromelysin 3)	6.27
HPSE	Heparanase	5.87
HRAS	V-Ha-ras Harvey rat sarcoma viral oncogene homolog	5.36
SYK	Spleen tyrosine kinase	4.49
IL18	Interleukin 18 (interferon-gamma-inducing factor)	4.16
CXCR4	Chemokine (C-X-C motif) receptor 4	3.98
RPSA	Ribosomal protein SA	3.43
IL1B	Interleukin 1, beta	3.32
FGFR4	Fibroblast growth factor receptor 4	2.89
DENR	Density-regulated protein	2.56
ETV4	ETS variant 4	2.49
MMP10	Matrix metallopeptidase 10 (stromelysin 2)	2.16
Down-regulated gene		
CTBP1	C-terminal binding protein 1	0.42
RORB	RAR-related orphan receptor B	0.41
FAT	FAT tumor suppressor homolog 1 (Drosophila)	0.39
BRMS1	Breast cancer metastasis suppressor 1	0.34
CST7	Cystatin F (leukocystatin)	0.24
CD82	CD82 molecule	0.20
TNFS10	Tumor necrosis factor (ligand) superfamily, member 10	0.16
RB1	Retinoblastoma 1 (including osteosarcoma)	0.13
TRPM1	Transient receptor potential cation channel, subfamily M, member 1	0.12

Among a total of 84 genes analyzed, the relative expression of 14 genes was up-regulated (by more than twofold) in response to estrogen, while 9 genes were downregulated (by less than 0.5-fold)

**Fig. 5:**
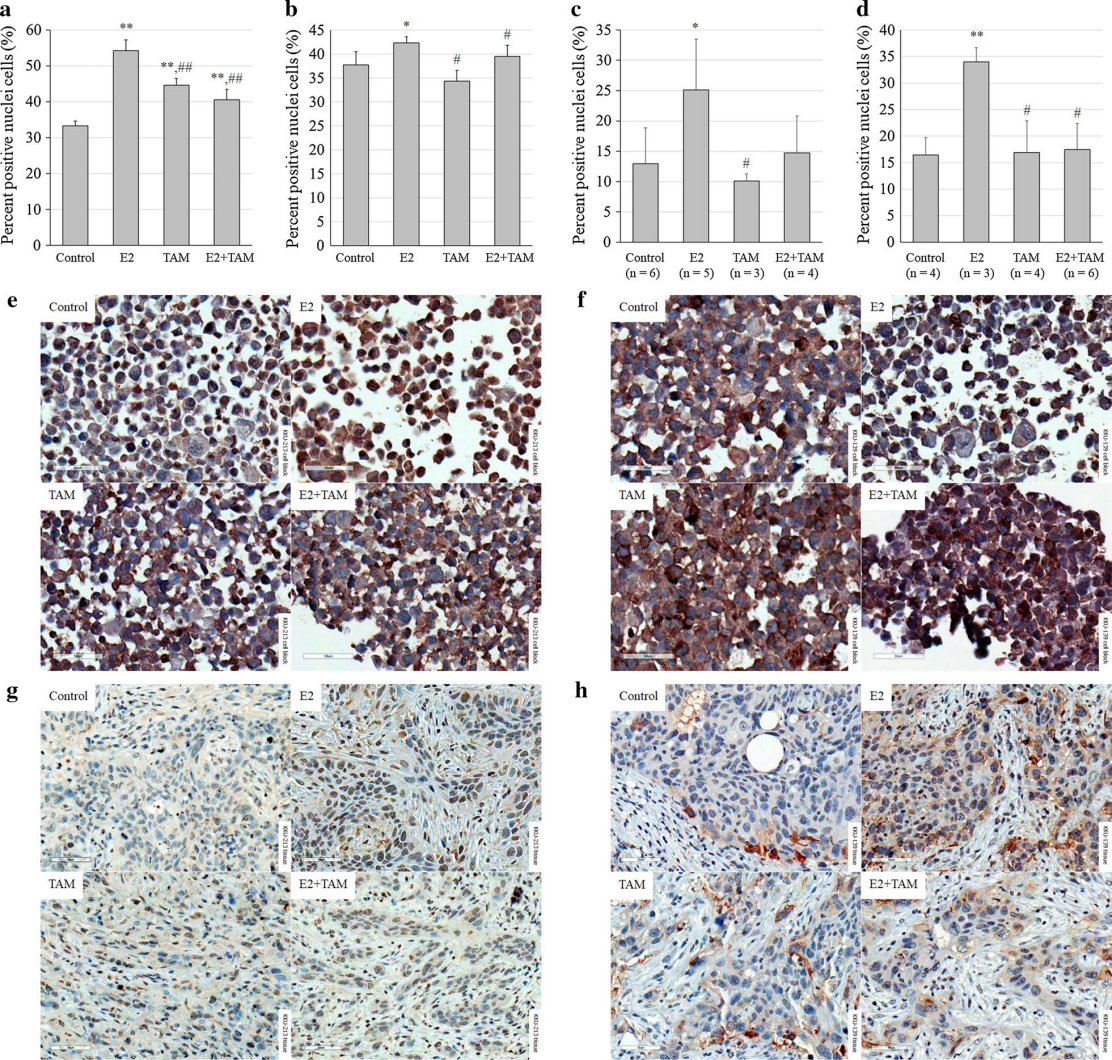
Relative nuclear expression of ETV4 protein in CCA cell lines treated with E2 and/or TAM compared to untreated control: **a** KKU-213 treated in vitro; **b** KKU-139 treated in vitro; **c** KKU-213 xenografted tumor masses; **d** KKU-139 xenografted tumor masses; **e** ETV4 staining of KKU-213 cell block; **f** ETV4 staining of KKU-139 cell block; **g** ETV4 staining of KKU-213 xenografted tumor masses; **h** ETV4 staining of KKU-139 xenografted tumor masses. For in vitro experiments, analyses were performed from five different fields. Number of mice (n) in each group was determined in diagrams. Cells with dark brown nuclei were counted as positive, in contrast to pale blue nuclei which were not considered positive. Original magnification of all analyzed images was 400×. Scale bar = 50 µM. Symbol * and ** determined statistically significant difference compared to untreated control group with *P* < 0.05 and < 0.001, respectively. Symbol # and ## determined statistically significant difference compared to E2 treated group with *P* < 0.05 and < 0.001, respectively

These differences were also reflected in the relative percentages of ETV4-positive stained nuclei in blocks of cells from different treatment groups. The percentage of positive cells in CCA cell lines treated in vitro with E2 was significantly higher in comparison with the other treatment groups, while the number of positive cells was reduced in the TAM treatment group (Fig. 5a, b). Comparing the xenografted tumors from both cell lines, the ETV4 staining was highest in tissues extracted from E2-treated mice (Fig. 5c, d). Representative pictures of stained nuclei from each group are shown in Fig. 5e–h.

### Role of ETV4 in E2-induced CCA cell progression

**Fig. 6:**
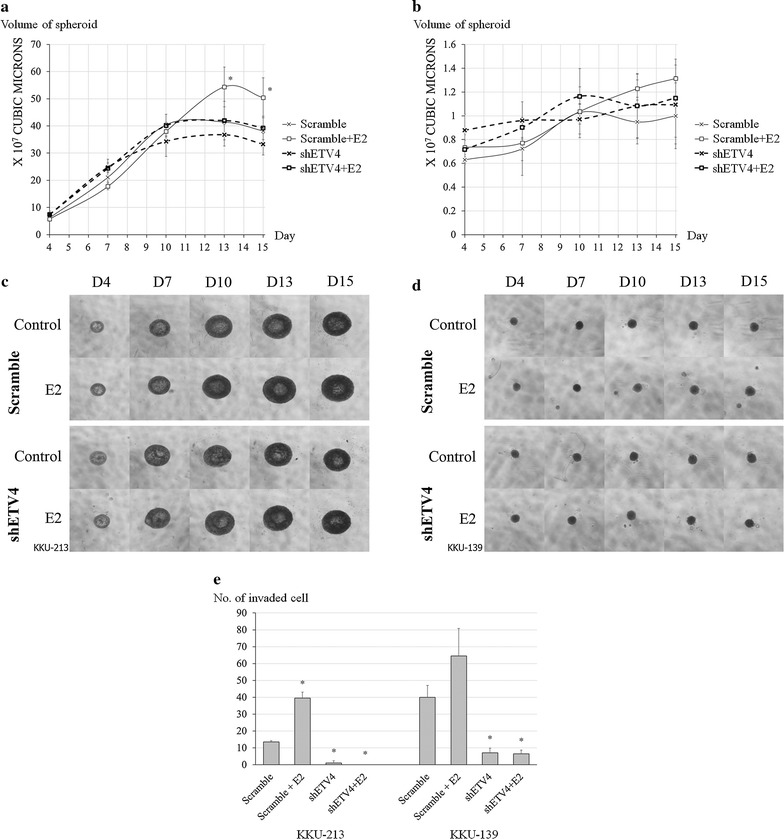
*In vitro* effect of estrogen on tumorigenesis properties of ETV4-knockdown CCA cell lines. Spheroid proliferation assays were performed as triplicated experiments and in vitro invasion experiments were performed as duplicated experiments. **a** and **b** Estrogen-stimulated spheroid growth of scramble- and ETV4-knockdown. **a** KKU-213 and **b** KKU-139 CCA cell lines in a 3D system. Arrows indicate the variables compared for statistical significance. **c** and **d** Representative pictures of spheroids of scramble- and ETV4-knockdown of **c** KKU-213 and **d** KKU-139. **e** In vitro invasion assay of scramble- and ETV4- knockdown of KKU-213 and KKU-139 CCA cells stimulated by E2. Symbol * determined statistically significant difference compared to scramble without E2 treatment with *P* < 0.05

Transfection with a shRNA plasmid against ETV4 (shETV4) and carrying GFP was carried out in accordance with manufacturer’s instructions. The best strands of shETV4 for each cell line were selected by real time PCR, based on their knockdown efficacy in both cell lines. Effectiveness of knockdown procedure in both cell lines was shown in Additional file 1: Figure S2. The role of ETV4 on cell proliferation was also investigated using 3D tumor spheroid-based assay. E2 did not increase the volume of spheroids containing either of the shETV4-transfected cell lines (Fig. 6a, b). An apparent E2 could increase proliferation in both tumor spheroids containing scramble-transfected cells but statistical significance was shown in only KKU-213 cells. Representative spheroids for all conditions are depicted in Fig. 6c, d. E2 also had an inductive effect on the invasiveness of both CCA cell lines that had undergone scramble treatment. Knockdown of ETV4 introduced marked loss of invasive ability in both CCA cells when compared to scramble treatment and E2 could not correct this effect (Fig. 6e).

## Discussion

Estrogen has been reported to stimulate proliferation of cholangiocytes and has been considered a factor in the pathogenesis of biliary tree disorders [17–21]. Other studies have suggested that bile duct obstruction is a cause of the high levels of serum estrogen in CCA patients and is a consequence of impairment of enzymes which convert estrogen metabolites [8–10]. Our previous study showed that estrogen can stimulate CCA cell proliferation and invasion in vitro [9]; this finding has been confirmed in this study. This study also demonstrated stimulation of KKU-213 and KKU-139 tumor growth by E2, both in an in vivo xenograft model and in 3D cultures. Both KKU-213 and KKU-139 could produce trace amount of E2 which lower than normal level in human male (0.037–0.147 nM) [35] and might not interfere the experiments. Both cell lines were demonstrated the expression of ERα which lower than MCF-7 but higher than MDA-MB-231 and KKU-213 showed higher than KKU-139 (Additional file 1: Figure S1). Moreover, KKU-213 was better than KKU-139 at forming spheroids in 3D culture, while KKU-139 was more successful than KKU-213 as a xenograft (data not shown) despite smaller tumor sizes, and this was observed even in E2 treated mice (Fig. 3). These results suggest that the two CCA cell lines may have different properties, but both were still stimulated to proliferate in response to E2.

There are some caveats to the present in vivo study. Some of the animals developed tumor metastasis, despite having a markedly small primary site (data not shown). This phenomenon occurred randomly, in all treatment conditions, and therefore may have arisen from the procedure of subcutaneous injection itself. Accordingly, samples from these animals were not included in the analysis. Nevertheless, the remaining samples revealed statistically significant differences between treatment groups. In addition, it was not always possible to measure the serum estrogen level from every animal because some samples had marked hemolysis and had to be discarded. Only male animals were used but the results showed raised serum estrogen in both E2-, and E2 with TAM-treated groups, therefore the oral treatment of male mice was considered sufficient.

The estrogen inhibitor TAM produced a strong inhibitory effect on the action of E2 in both the in vitro and the in vivo models. TAM is the first selective estrogen receptor modulator (SERM), which acts as an antagonist, to have been successfully used as a hormonal treatment of breast cancer, most notably in ER-positive breast cancer [36]. Other well-known SERMs used recently to treat cancers include toremifene, raloxifene, arzoxifene, among others [36]. Unlike estrogens, some members of the SERM family, such as TAM, show a tissue-selective pharmacological effect, e.g. acting as estrogen agonists in the skeletal and cardiovascular systems, while acting as antagonists in breast cancer [36]. Since the use of TAM in ER positive cancers, such as breast and ovarian carcinomas, is well established, CCA, which is also an ER positive cancer [9, 17], may be responsive to TAM as well. Since 1997, there have been reports of a dose-dependent TAM inhibition of CCA cell growth, as well as inhibition of tumor growth in a mouse xenograft model [37], and reports of the use of TAM in other systems to enhance the therapeutic effect of cytotoxic drugs such as adriamycin, mitomycin, vindesine [38, 39] and gemcitabine [40]. Possible mechanisms by which TAM decreases CCA tumorigenesis have been explored and may include interferon gamma [41] and calmodulin modulation [42]. Clearly, the concept that TAM may offer an alternative or adjunctive treatment for CCA has been generating interest [43].

Estrogen stimulates cells by binding to its receptors, ERs, which function as ligand-activated transcription factors upon binding to their cognate DNA sequences, EREs [44]. We therefore screened for expression of selected estrogen-induced genes, using a metastasis PCR array set to define an expression profile. This led to focus upon ETV4 because it was: an up-regulated gene; a transcription factor activating both metastasis and proliferation, and; in a gene structural location associated with estrogen function, while the rest genes would be the useful data for further study. Furthermore, in many studies, the roles of ETV4 in various types of tumor have been reported [22–28]. The overexpression of ETV4 impacts on many aspects of tumorigenesis including cell proliferation, invasion and metastasis [23, 25, 26]. Regulation of ETV4 expression by estrogen has been supported, not only by its genetic structure [29], but also by the observation that TAM can down-regulate its expression [45]. In this study, the modulated expression of ETV4 in CCA cells was confirmed by RT-real time PCR and immunocytochemistry/immunohistochemistry, both in two tumor cell lines and in mouse tumor tissues that were stimulated by E2, and inhibition by TAM was demonstrated. Because ETV4 is a transcription factor, functioning primarily within the cell nucleus [22], we stained nuclei and measured intra-nuclear expression of the protein by software to determine the stimulation of ETV4 expression by E2 and efficacy of knockdown by shRNA. Therefore, our results were showing that ETV4-knockdown KKU-213 CCA cells lost the ability to be stimulated by E2 were consistent with a role of ETV4 in estrogen-induced CCA proliferation (Fig. 6a). However, in KKU-139 cells which showed less ability of spheroid forming did not present the significant difference of E2 induced growth between scramble and ETV4 knockdown conditions (Fig. 6b). Moreover, ETV4 also showed critical role in invasiveness of both CCA cells as the knockdown cells had almost loss of the invasive ability that could not be rescued by E2 (Fig. 6e). In a genetically engineered mouse model of metastatic prostate cancer, combined activation of PI3-kinase and Ras signaling could activate ETV4 and leading to promote metastasis [46]. In addition, several studies have suggested that the pathways by which ETV4 promotes metastasis in various type of cancers include extracellular signal-regulated kinases signaling associated with matrix metalloproteinase enzyme [24, 47, 48]. Moreover, according to that ETV4 functions as a transcriptional factor, one of an interesting target for ETV4 is indoleamine 2,3-dioxygenase (IDO), an enzyme that involved in progression of many type of cancer [49, 50]. For examples, IDO had been reported the roles in immune escape [50], tumor growth [51] and angiogenesis [52], which could determine prognosis of the patients. These evidences reflected the role of ETV4 in estrogen-induced CCA progression. Taken all together, ETV4 could be considered as an intermediate molecule in E2 stimulated CCA progression in this study.

## Conclusion

This experiment demonstrated the strength of estrogen to promote CCA progression in both proliferation and invasion properties. ETV4 was considered as an intermediate in this process. Tumor growth promotion had been confirmed by mouse xenograft model and oral supplement of TAM showed to inhibit this effect. Therefore, TAM can be considered as an adjuvant for CCA treatment in clinical use.

## Additional file

**Additional file 1: Table S1.** Primer sequences for gene expression analysis of ERs. **Figure S1.** Relative expression of ERs in CCA cells compared to MCF-7 and MDA-MB-231 breast cancer cells: (A) ER-α; (B) ER-β. **Figure S2.** Efficacy of shETV4 plasmid transfection and knockdown: (A) Transfection efficacy determined by expression of GFP and visualized under fluorescence inverted microscope with 40x original magnification, cell type and conditions were labelled in the picture; (B) Knockdown efficacy in transfected cells determined by RT-real time PCR and compared to parental cell mRNA.

## Abbreviations

3D: three dimentional
CCA: cholangiocarcinoma
E2: 17β-estradiol
TAM: tamoxifen
ETV4: E26 transformation-specific variant 4
PEA3: polyomavirus enhancer activator 3
ERs: estrogen receptors
EREs: estrogen response elements
shETV4: short hairpin RNA for ETV4
SERM: selective estrogen receptor modulator
