# Supplementary material for: Increased ETV4 expression correlates with estrogen-enhanced proliferation and invasiveness of cholangiocarcinoma cells
Source: Cancer Cell Int. 2018 Feb 20;18:25. doi: 10.1186/s12935-018-0525-z (PMC5819217; doi:10.1186/s12935-018-0525-z)
Supplement: Supplementary file 1 — Additional file 1: Table S1. Primer sequences for gene expression analysis of ERs. Figure S1. Relative expression of ERs in CCA cells compared to MCF-7 and MDA-MB-231 breast cancer cells: (A) ER-α; (B) ER-β. Figure S2. Efficacy of shETV4 plasmid transfection and knockdown: (A) Transfection efficacy determined by expression of GFP and visualized under fluorescence inverted microscope with 40x original magnification, cell type and conditions were labelled in the picture; (B) Knockdown efficacy in transfected cells determined by RT-real time PCR and compared to parental cell mRNA. [file 12935_2018_525_MOESM1_ESM.pdf]

Table S1 Primer sequences for gene expression analysis of ERs

Gene	Accession no.		Primer sequence (5`→ 3`)	Size (bp)
$ER\alpha$	NM_ 000125.3	F	GAATCTGCCAAGGAGACTCGC	151
		R	ACTGGTTGGTGGCTGGACAC	
$ER\beta$	NM_ 001437.2	F	TGTCTGCAGCGATTACGCA	145
		R	GCGCCGGTTTTTATCGATT	
36B4	NM_001002.3	F	CTTCCCACTTGCTGAAAAG	168
		R	CCAAATCCCATATCCTCGT	

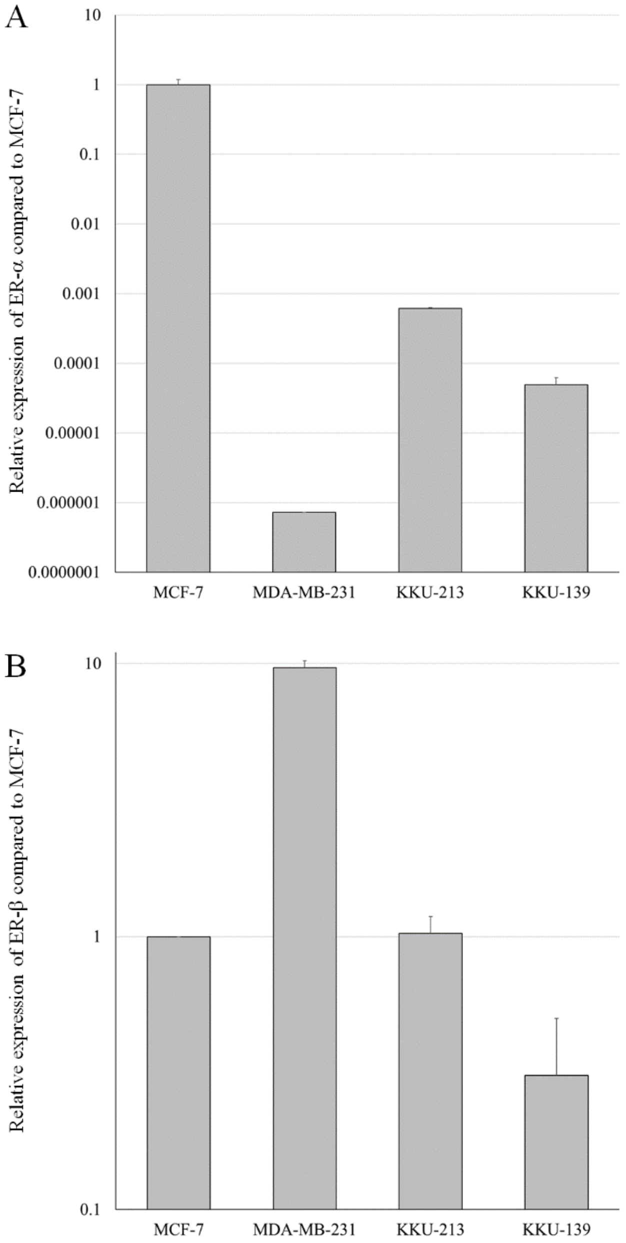

Figure S1 Relative expression of ERs in CCA cells compared to MCF-7 and MDA-MB-231 breast cancer cells: (A) ER-α; (B) ER-β.

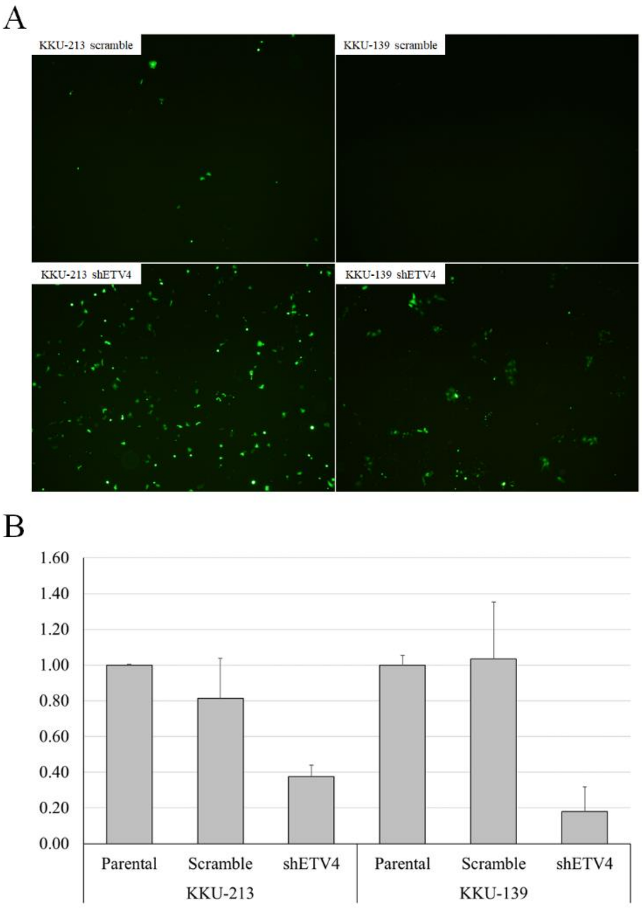

Figure S2 Efficacy of shETV4 plasmid transfection and knockdown: (A) Transfection efficacy determined by expression of GFP and visualized under fluorescence inverted microscope with 40x original magnification, cell type and conditions were labelled in the picture; (B) Knockdown efficacy in transfected cells determined by RT-real time PCR and compared to parental cell mRNA.
